# The pivotal role of irradiation‐induced apoptosis in the pathogenesis and therapy of medulloblastoma

**DOI:** 10.1002/cnr2.2048

**Published:** 2024-04-10

**Authors:** Seidu A. Richard

**Affiliations:** ^1^ Department of Medicine Princefield University Ho Ghana; ^2^ Institute of Neuroscience, Third Affiliated Hospital Zhengzhou University Zhengzhou China

**Keywords:** apoptosis, DNA, irradiation, medulloblastoma, radioresistance, radiosensitivity

## Abstract

**Background:**

Medulloblastoma (MB) is a rare primitive neuroectodermal tumors originating from the cerebellum. MB is the most common malignant primary brain tumor of childhood. MB originates from neural precursor cells in distinctive regions of the rhombic lip, and their maturation occurs in the cerebellum or the brain stem during embryonal development. Also, apoptosis is a programmed cell death associated with numerous physiological as well as pathological regulations.

**Recent Findings:**

Irradiation (IR)‐induce apoptosis triggers cell death, with or without intervening mitosis within a few hours of IR and these share different morphologic alteration such as, loss of normal nuclear structure as well as degradation of DNA. Moreover, MB is strikingly sensitive to DNA‐damaging therapies and the role of apoptosis a key treatment modality. Furthermore, in MB, the apoptotic pathways are made up of several triggers, modulators, as well as effectors. Notably, IR‐induced apoptotic mechanisms in MB therapy are very complex and they either induce radiosensitivity or inhibit radioresistance leading to potential effective treatment strategies for MB.

**Conclusion:**

This review explicitly explores the pivotal roles of IR‐induced apoptosis in the pathogenesis and therapy of MB.

## 1 INTRODUCTION

Medulloblastoma (MB) is a rare primitive neuroectodermal tumors (PNET) originating from the cerebellum and they are made up of 1.7% of all primary central nervous system (CNS).^1^, ^2^ Notably, MB compose of different variants and it is the most common malignant primary brain tumor of childhood.^2^ MB originates from neural precursor cells in distinctive regions of the rhombic lip, and their maturation occurs in the cerebellum or the brain stem during embryonal development.^3^, ^4^, ^5^ MB is classified into four biological subgroups such Wingless (WNT), Sonic Hedgehog (SHH), Group 3, and Group 4 based on microarray as well as genomic sequencing technologies.^6^, ^7^, ^8^ Currently, the standard treatments for MB comprises of surgery, chemotherapy as well as cerebrospinal irradiation (IR) which cumulatively augments the 5‐year survival prognosis rate to approximately 60%.^6^, ^7^, ^9^, ^10^

Notably, apoptosis is a programmed cell death associated with numerous physiological as well as pathological regulations and apoptotic pathways are made up of diverse triggers, modulators, as well as effectors.^11^ It is worth noting that apoptosis is primarily initiated via either extrinsic via the ligand/receptor mediated or intrinsic via the mitochondrial signaling pathways.^12^ Remarkably, IR‐induce apoptosis triggers cell death, with or without intervening mitosis within a few hours of IR and these share different morphologic alteration such as, loss of normal nuclear structure as well as degradation of DNA.^13^ Interestingly, most MBs are strikingly sensitive to DNA‐damaging therapies and the role of apoptosis a key treatment modality.^13^

In addition, one of the primary clinical challenges associated with MB therapy is its proclivity to spread throughout the neuraxis although the disease is often localized thus, IR therapy often spread to the entire neuraxis.^14^ Interestingly, IR to the entire neuraxis results in 60% long‐term survival.^14^, ^15^ Intriguingly, anatomical location, microenvironment and genetic modifications of these tumors differ from human tumors in their in vivo setting and all of these parameters affect the tumor's response to therapy. This review focus mainly on the pivotal roles of IR‐induced apoptosis in the pathogenesis and therapy of MB (Table 1).

**TABLE 1:** Show a summary of the target agents and irradiation induce apoptosis therapeutic mechanisms.

Target agent	Irradiation induce apoptosis therapeutic mechanisms	Reference
p53	A single mutation in the apoptosis pathway was sufficient to trigger IR resistance via apoptosis in MB tumors with intact p53	4, 33
PKB	Inhibition of PKB triggered a significate decrease in cell proliferation and increased apoptosis, with a decrease phosphorylation of downstream effectors of PKB such as ERK1/2 and AKT in MB following IR via apoptosis	27
Celecoxib (COX‐2)	Radiosensitizing effects of celecoxib on MB‐CD133^+^ cells, in vitro and in vivo, augmented radiosensitivity as well as IR‐induced apoptosis	110
hMSCs	The benefits of engineered hMSCs in a xenograft mouse model of MB for a TRAIL‐sensitive cell line, and the sensitizing effect of HDAC inhibitor MS‐275 to stem cell‐delivered TRAIL on a TRAIL resistant cell line was established via apoptosis	9
uPAR and MMP‐9	Concurrent silencing of uPAR as well as MMP‐9 using RNAi vectors acted as a potential vector for MB gene therapy in combination with IR treatment via apoptosis	48
PCNA	PCNA act as a monoubiquitinate via E3 ligase RNF8 in IR‐induced MB cells leading to low radiosensitivity in MB cells	48
Onvansertib	Onvansertib in combination with IR triggered a total tumor regression of MB xenografts via apoptosis	67
*Ptch1/SMO/SUFU/GLI* axis	Interfering with the *Ptch1/SMO/SUFU/GLI* axis in the hedgehog pathway with small molecules has been implicated as a promising therapy for SHH MBs via apoptosis	27
Veliparib	Veliparib in combination with IR significantly augmented intratumoral apoptosis on immunohistochemistry staining, signifying that veliparib augmented IR induced MB cell death in vivo	129
RBM5‐AS1	Knockout of RBM5‐AS1 triggered sensitization of DAOY xenograft tumors to IR, which was associated with augmented apoptosis	50
NPI‐0052	A combination of IR and proteasome inhibitors in inducing apoptosis in MB tumor cells resulting in tumor reduction	51
STAT3	STAT3 blockers such as LLY17 as well as LLL12B were capable of augmenting the blockade activity of IR in human MB cells via apoptosis Both Cyclin D1 and Survivin secretory levels were reduced after combination treatment with LLY17 or LLL12B with IR exposure and the induction of cell apoptosis as well as cleaved Caspase‐3 were augmented	52
p21 and NSCs	Deficient of p21 was linked to an obvious radioresistance in NSCs, whereas p21 secretion n correlates with a strong apoptotic response in fate‐restricted PCs	16
DNA‐PKcs	Loss of DNA‐PKcs function blocked MB growth in the *Ptch1* ^*+/−*^ mouse model via apoptosis following IR exposure DNA‐PKcs blocker NU7441 radiosensitizes human MB cells in vitro via apoptosis	13

Abbreviations: AS1, antisense RNA1; COX‐2, cyclooxygenase 2; DNA‐PKcs, DNA‐dependent protein kinase; IR, irradiation; MB, medulloblastoma; MMP, matrix metallopeptidase; NPI‐0052, marizomib or salinosporamide A; PKB, protein kinase B; PCNA, proliferating cell nuclear antigen; PCs, progenitor cells; *Ptch1*, protein patched homolog 1; RBM5, RNA binding motif protein 5; RNF8, RING finger protein 8; SHH, Sonic Hedgehog; SMO, smoothened; STAT, signal transducers and activators of transcription; TRAIL, TNF‐related apoptosis‐inducing ligand; uPAR, urokinase‐type plasminogen activator receptor.

The “Boolean logic” was used to search for article on the subject matter in PubMed and PubMed central as well as google scholar with search term like MB and/or IR‐induced apoptosis mechanisms such as DNA‐damage, signaling, immune modulation, hormonal imbalances, and therapeutic modalities. In addition, data on the categorization of MB was searched and discussed. Studies involving both humans and animals as well as both clinical research and basic research were critically reviewed. Articles that did not report or discuss interrelations between MB and IR‐induced apoptosis mechanisms were excluded from this review.

## 2 BRIEF CATEGORIZATION OF MEDULLOBLASTOMA

MB arises from neural precursor cells (NPCs) in distinctive zones of the rhombic lip, from where they grow into the cerebellum and/or the brain stem during embryonal development.^3^, ^5^ Specifically, NPCs of the ventricular zone (VZ) as well as the external granule layer (EGL) are precisely the cells of origin of MB.^16^ Notably, genetic, gene‐regulatory, or epigenetic aberrations, which blocks the normal neuronal or glial differentiation have been implicated in the initiation of tumor formation.^4^, ^5^ Remarkably, based on microarray and genomic sequencing technologies, MB has been categorized into four biological subgroups such as WNT, SHH, Group 3 as well as Group 4.^6^, ^7^, ^8^ Experimentally, cell lines such as D283, D425, UW288, as well as UW426 correspond to group 3/4, group 3, WNT‐activated, as well as SHH‐activated MB subtype, respectively.^17^

Interestingly, the WNT as well as SHH MB subgroups are predominantly obtained via mutations to enable them modulate the WNT as well as SHH signaling pathways, respectively.^7^ In addition, the WNT as well as SHH MBs have negligible similarity with other subgroups because they are distinctly separable across the majority of transcriptional as well as methylation profiling analyses.^7^ On the other hand, although the genetics as well as biology underlying Group 3 and 4 MB is unspecific, the transcriptomes of Group 3 and 4 MB are more analogous to each other because numerous cytogenetic features like isochromosome 17q (i17q) have been identified in both groups.^6^, ^8^ Additionally, isochromosome 17q which was linked to cell division protein kinase 6 (CDK6) as well as n‐MYC augmentation was detected in about two‐thirds of group 4 MB patients.^18^

Genetically, MB is categorized into WNT‐activated, SHH‐activated as well as TP53‐mutant, SHH‐activated and TP53‐wild‐type, as well as non‐WNT and non‐SHH groups.^19^ Notably, amalgamation of multiple molecular data bases also refined stratification MB into as many as 13 subtypes which includes WNT; SHHα, β, γ, or δ; and G3 or G4 subtypes I–VIII.^20^, ^21^ Interestingly, these subtypes exhibit diverse responses to current therapy, with the worse survival rates for MYC‐amplified G3 such as G3γ or G3, G4 or subtype II also known as G3‐II and TP53‐mutated SHH (SHHα) or outstanding survival for WNT MB.^20^, ^21^ In addition, the long‐term survival rate was up to 90% in the rare WNT subgroup, and typically approximately 50% in other subgroups, with an intermediate prognosis in Group 4 as well as poorest in Group 3 patients.^6^, ^22^

Notably, approximately one‐third of all MBs exhibits abnormal stimulation of the SHH signaling pathway, a developmental axis via tumor suppressor gene like protein patched homolog 1 (*Ptch1*) usually induce a blockade effect and augmentation of GLI1 or GLI2 as well as the deletion of *Ptch1* are commonly detected in the SHH subgroup.^23^, ^24^ In addition, somatic catenin beta 1 (CTNNB1) mutations as well as chromosome 6 loss are frequent in the WNT subgroup.^25^ Furthermore, abnormal MYC augmentation have been observed in about 17% of patients with group 3 MB and this reflects an essential feature of this subgroup.^26^ Remarkably, the secretory level of protein kinase B (PKB) was apparently elevated in all MB subgroups compared to normal brain tissue, and elevated PKB secretion correlated with a poor prognosis in SHH, group 3 as well as group 4 MBs.^27^

## 3 IRRADIATION INDUCED APOPTOSIS IN MB

X‐rays or x‐irradiation (x‐IR) are photons emitted from the electron shells surrounding the atomic nucleus. The term x‐IR often refer to IR coming from the atomic nucleus. Gamma irradiation (γ‐IR) is a form of IR that utilize gamma rays. FLASH radiotherapy (FRT) is a new technique use to delivery of ultra‐high dose rate IR to a target.^28^ Currently no studies have utilized FRT to investigate the roles of IR‐induced apoptosis in the pathogenesis and therapy of MB. Thus, studies are warranted in this direction.

**FIGURE 1:**
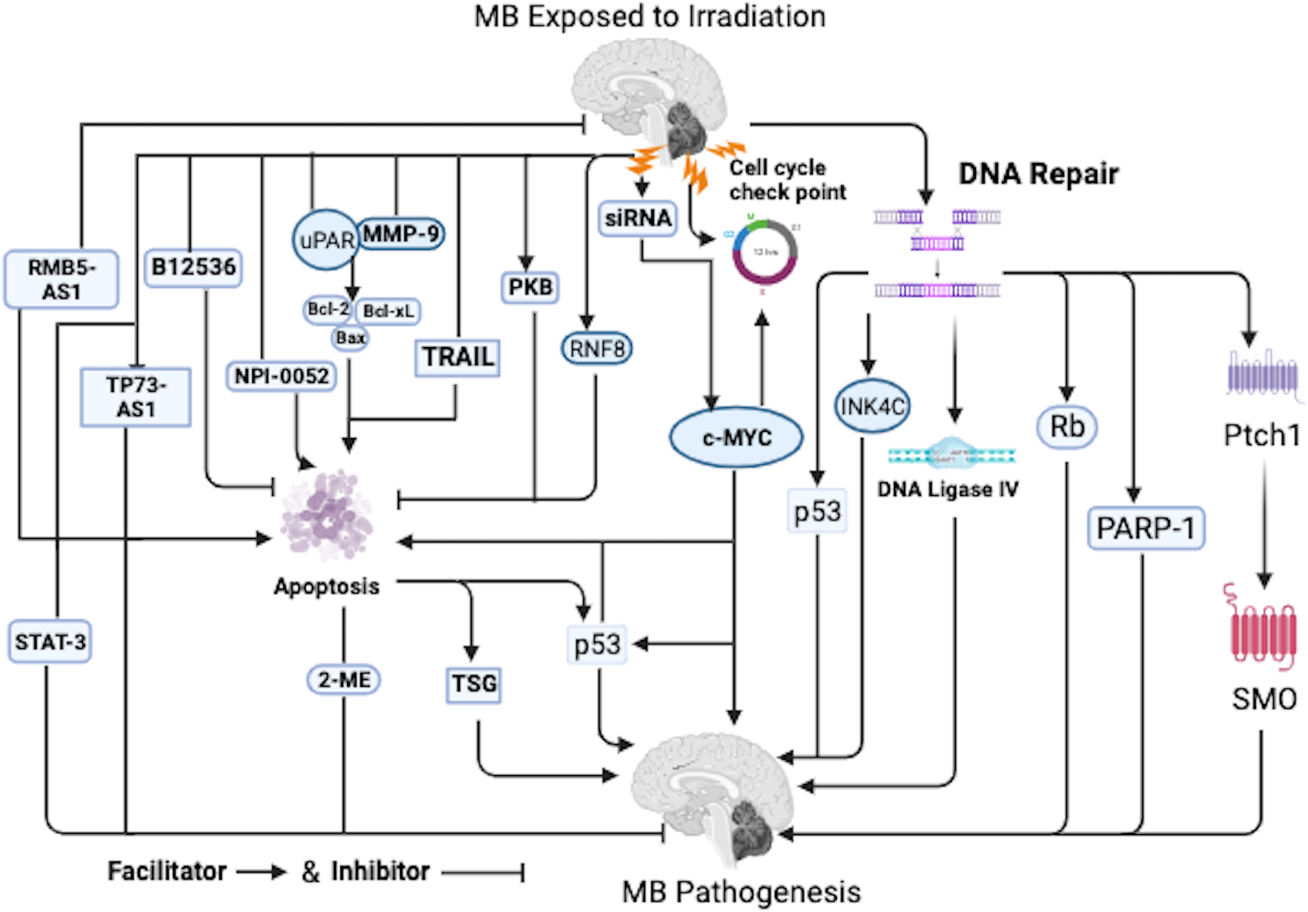
Shows the irradiation induced apoptosis mechanisms in MB. Refer to the text for detailed explanations. Furthermore, all different variants of MB are collective term MB in the diagram. Bax, Bcl‐2‐associated X protein; Bcl2, B‐cell lymphoma 2; INK4C, INhibitors of CDK4C; MMP, matrix metallopeptidase; NPI‐0052, marizomib or salinosporamide A; PARP‐1, poly (ADP‐ribose) polymerase‐1; PKB, protein kinase B; *Ptch1*, protein patched homolog 1; Rb, retinoblastoma protein; RBM5, RNA binding motif protein 5; RNF8, RING finger protein 8; siRNA, small interfering RNA; SMO, smoothened; STAT, signal transducers and activators of transcription; TP73‐AS1, tumor protein P73 antisense RNA 1; TRAIL, TNF‐related apoptosis‐inducing ligand; TSCs, tumor stem cells; uPAR, urokinase‐type plasminogen activator receptor.

It is worth noting that several permutations in targeted deletions in genes regulating, apoptosis via cell cycle checkpoints as well as DNA repair, such as *Ptch1*, retinoblastoma protein (Rb), DNA Ligase IV, poly (ADP‐ribose) polymerase‐1 (PARP‐1) as well as cyclin‐dependent kinase 4 inhibitor C (INK4C) together with p53 loss have detected in murine MB with IR and an alternative method to complement *Ptch1* mutation with IR was also detected (Figure 1).^29^, ^30^, ^31^ Interestingly, hypermethylation of gene promotors of tumor suppressor genes (TSG), which triggered their inactivation, unhindered proliferation as well as inhibition of apoptosis was detected in 70%–90% of primary MBs (Figure 1).^4^

Notably, blocked smoothened (SMO) was unbound as well as downstream SHH targets genes upon binding of SHH to its receptor *Ptch1* in the canonical SHH signaling (Figure 1).^16^ Interestingly, IR of *Ptch1* ^+/−^ mice which are knockout of *Ptch1* mice during postnatal days 1–4 revealed that granule progenitor cells (PCs) are extremely proliferative as well as severely augmented the incidence of MB.^29^, ^31^ In addition, using conditional *Ptch1* knockout, it was observed that both stem cells (SCs) as well as PCs cells are origin for MB, and these cells have been implicated in the rate of tumor progression.^32^

Intriguingly, p53 inactivation correlated with reduced levels of IR‐induced apoptosis in MB cell lines as well as poor prognosis (Figure 1).^33^ It is worth noting that, a single dose of IR exposed to MB induced apoptosis in the primary tumor cells and cell cycle arrest in the stem cell segment in a p53‐dependent manner.^34^ Notably, p53 wild‐type cell line was more sensitive to x‐IR than the p53 mutated cell line, indicating that p53 mutations triggered the advancement of aggressive types of MB.^35^ In addition, it was observed that p53 induction after IR was typically limited to the EGL, the same region that undergoes apoptosis in response to genotoxic stress, signifying that the apoptotic process transpiring in IR cerebellum was mainly driven by stimulation of p53 during immunohistochemical analysis.^29^

Furthermore, a significantly reduced level of p53 in P1 was observed after IR expose compared to IR expose to P10 mice cerebellum during immunoblot as well as immunohistochemical analysis.^29^ Additionally, a correlation between the delayed and reduced p53 secretion and the resistance to IR induced cell killing was detected in cerebellar granule neuron progenitors (CGNPs) at P1, signifying that augmented IR‐induced apoptosis influenced cerebellar development progresses.^29^ Notably, knockout of RING finger protein 8 (RNF8) an E3 ubiquitin ligase blocked the effect of IR‐induce apoptosis in MB cells (Figure 1).^36^ In addition, BI2536, a potent and selective inhibitor of polo‐like kinase 1 (PLK1) blocked MB cell proliferation, self‐renewal, cell‐cycle progression, as well as induced apoptosis following IR (Figure 1).^37^

Furthermore, blockade of PKB decreased the proliferation as well as induced apoptosis in two MB cell lines in vitro (Figure 1).^27^ Comparatively, the utmost proportion of apoptotic cells were detected 48 h post IR in both DAOY as well as D283MED cells with the apoptosis values three times greater in D283MED cells.^38^ It is worth noting that elevated c‐MYC mRNA secretion, c‐MYC gene augmentation, as well as low‐level copy alterations of c‐MYC have been implicated as unfavorable prognosis factors for MB following IR (Figure 1).^39^, ^40^ Interestingly, c‐MYC is capable of driving both proliferation and apoptosis because it is a regulator of S‐phase entry, proliferation, as well as differentiation (Figure 1).^41^, ^42^ Notably, downregulation of c‐MYC was capable decreasing the sensitivity of MB cells such as D341, D425, and DAOY to IR when small interfering RNA (siRNA) was used to transiently inhibit c‐MYC expression in these cell lines (Figure 1).^43^

Interestingly, IR‐induced apoptosis was only observed in DAOY M2 cells.^44^ Besides, DAOY cells engineered to oversecrete c‐MYC made p53 mutant DAOY cells susceptible to the stimulation of apoptosis (Figure 1).^45^ In addition, it was observed that 2‐methoxyestradiol (2‐ME) blocked the growth of DAOY cells via induced apoptosis (Figure 1).^1^ Notably, tumor necrosis factor (TNF)‐related apoptosis‐inducing ligand (TRAIL) has also been implicated to induce apoptosis in some MB cell lines following IR.^46^, ^47^ Interestingly, cell‐based delivery of TRAIL was extremely efficient in orthotopic MB models because of the tumoritropic capabilities of SCs as well as the persistent expression of TRAIL on site of the tumors.^48^

It is worth noting that blockade of both urokinase‐type plasminogen activator receptor (uPAR) and matrix metallopeptidase (MMP)‐9 secretion restrained proliferation and induces apoptotic cell death in MB during functional analyses.^49^ In addition, blocked of uPAR and MMP‐9 altered downstream signaling molecules resulting in transcriptional blockade of anti‐apoptotic molecules as well as stimulation of MB cells towards apoptosis.^49^ Notably, combining IR treatment to uPAR, MMP‐9 as well as a combination of uPAR and MMP‐9 transfected MB cells exhibited a greater efficiency in inducing apoptosis (Figure 1).^49^

Interestingly, the induced apoptotic stimulated via downregulation of uPAR and MMP‐9 inhibited the function of anti‐apoptotic B‐cell lymphoma (Bcl)‐2 as well as Bcl‐xL molecules and triggered pro‐apoptotic Bcl‐2‐associated X protein (Bak) in MB cells following IR exposure (Figure 1).^49^ In addition, knockout of non‐coding RNA (lncRNA)‐tumor protein P73 antisense RNA 1 (TP73‐AS1) induced apoptosis as well as blocked cell proliferation and migration in MB cells following IR.^50^ Furthermore, lncRNA‐RNA binding motif protein 5‐antisense RNA1 (RBM5‐AS1) silencing augmented IR induced apoptosis in MB cells.^51^

Interestingly, over secretion of RBM5‐AS1 shielded MB cells from IR‐induced apoptosis (Figure 1).^51^ It is worth noting that NPI‐0052 induces blockade of proteasome activity with consequent apoptosis stimulation in MB cells following IR (Figure 1).^52^ Remarkably, signal transducers and activators of transcription (STAT)‐3 blockers such as LLY17 as well as LLL12B in combination with IR efficiently blocked cell viability, cell migration, invasion as well as tumor sphere growth and induce cell apoptosis of human MB cells (Figure 1).^53^

## 4 IRRADIATION AND DNA IN MB

Eukaryotic cells preserve genome integrity via DNA damage response (DDR) network, which triggers a sequence of signal transduction cascades resulting in DNA repair, apoptosis, as well as chromatin remodeling.^54^ Notably, DNA double‐strand breaks (DSBs) are detrimental DNA damages triggered by unrepaired or misrepaired DSBs resulting in genome reordering such as lethal mutations as well as chromosome aberrations which leads to genome instability, apoptosis, tumorigenesis, as well as immune deficiency following IR.^13^, ^36^ Remarkably, DNA impairments are sensed by the DDR, which is capable of transmitting signaling via a sequence of exceedingly well‐ordered responses to safeguard cells from damages.^55^ In addition, reactive oxygen species (ROS) and free radicals are generated which induce DNA damages following exposure to IR.^56^

Intriguingly, IR‐induced DNA damage is often repaired by cells through the homologous recombination (HR) as well as nonhomologous end‐joining (NHEJ) pathways, leading a decline in cell damage as well as IR sensitivity.^57^ Notably, in MB, DNA damage induced by IR extremely augmented development in newborn *Ptch* ^*neo67/+*^ mice in which the EGL was still proliferating.^31^ Interestingly, DDR triggered DNA damage repair via the modulation of cell cycle or apoptosis of cells upon activation of DSBs, which resulted in reduced sensitivity of MB cells to IR.^29^ Remarkably, timing of DNA damage was modulated when *Ptch* ^*neo67/+*^ mice bearing MB were exposed to IR. This made way for in vivo analysis of early changes in cerebellar signaling as well as the steps of the tumorigenic process from initial gene deregulation to MB development.^29^

Notably, DNA‐dependent protein kinase (DNA‐PKcs) driven DNA repair to block cell apoptosis is one of several mechanisms via which DNA‐PKcs mediated oncogenic behaviors.^58^, ^59^ Remarkably, low‐dose IR of mice with total loss of the NHEJ core factor DNA‐PKcs triggered high DSBs burden that augmented apoptosis of tumor initiating‐cells leading to lessened cancer frequency in vivo.^13^ In addition, loss of function of core factors such as DNA‐PKcs and Rad54 suppresses and promotes triggered *Ptch1*‐associated MB tumorigenesis after exposure to x‐IR in vivo.^60^

Moreover, DNA‐PKcs‐dependent p53 activation was key inhibitor of MB tumorigenesis in *Ptch1*+/− mice, because mice with disruption of other NHEJ core components such as DNA Ligase 4 (Lig4), X‐ray repair cross‐complementing protein 4 (XRCC4), Ku80 and Artemis developed MBs only in p53‐null experiments, signifying a stern dependence of tumorigenesis on the blockade of p53 surveillance mechanisms.^61^, ^62^ Furthermore, DNA‐PKcs genetic inactivation in *Ptch1* ^+/−^ mice bestowed IR hypersensitivity by triggering cell cycle arrest, p53 activation as well as apoptosis at exceedingly low IR doses, whereas sparing the developing cerebellum from the buildup of stress‐induced oncogenic DNA damage.^13^

Interestingly, it was observed that loss of *Ptch1* function facilitated IR‐induced MB tumorigenesis because all wild types tumors were characterized by loss of wild type *Ptch1* allele.^29^ Notably, p53 loss considerably facilitated the age of onset as well as incidence of MB in *Ptch1* heterozygotes because 95% of the *Ptch1* ^+/−^/p53^−/−^ mice developed MB by 12 weeks of age.^30^ It was further observed that ectopic secretion of RBM5‐AS1 inhibited IR‐induced DNA damage in MB cells signifying the pro‐survival capability of RBM5‐AS1 in MB cells.^51^ In addition, silencing of Sirtuin 6 (SIRT6) augmented IR‐induced DNA damage, which was analogous to the finding in RBM5‐AS1‐depleted cells.^51^ Intriguingly, induced secretion of SIRT6 reverses RBM5‐AS1 depletion‐induced radiosensitization as well as DNA damage response.^51^

Notably, RNF8 has been implicated in processing of DNA damage repair via the augmentation in the recruitment of modulatory proteins at the sites of DNA damage.^36^ Intriguingly, RNF8 was capable of recruiting numerous proteins like tumor protein p53‐binding protein 1 (53BP1), RAD51, as well as early type breast cancer 1 (BRCA1), at the sites of DNA damage via the modulation of ubiquitination of histone H2A as well as histone H2A family member X (H2AX) to accelerate DNA repair.^36^ In addition, abnormally secretion of RNF8 have been implicated in the disruption of transduction of DDR as well as DNA damage repair.^63^

It is worth noting that RNF8 was a crucial in facilitation as well as modulation of the DDR during IR exposure in human MB cells.^36^ Additionally, RNF8 was upregulated in MB cells and was also significantly elevated by γ‐IR exposure in a dose‐dependent manner. Moreover, knockout of RNF8 triggered blockade of survival as well as affect the apoptosis and cell cycle arrest of MB cells after IR exposure leading to augmentation in the sensitivity of MB cells to IR.^36^ Furthermore, RNF8 blockade expressively augmented the recruitment of γ‐H2AX at DNA damage sites in MB cells exposed to IR.^36^ Notably, proliferating cell nuclear antigen (PCNA) which is structurally as well as functionally preserved molecular medium has been implicated as coordinator for core DNA synthesis machinery.^36^

Interestingly, it forms a homotrimeric ring enclosing the DNA double helix to enable it triggers molecular medium that accelerate the protein–protein as well as protein‐DNA interactions transpiring at the replication fork.^64^ In addition, PCNA has been implicated as key target for E3 ubiquitin modification during the DDR signaling pathways.^65^ Remarkably, RNF8 triggered the recruitment of DNA damage repair‐associated proteins at the site of DNA damage by modulating the ubiquitination of PCNA.^36^ In addition, RNF8 knockout augmented the IR sensitivity of MB cells modulating the ubiquitination of PCNA. Thus, the secretion of RNF8 was upregulated after IR exposure, and silencing RNF8 lead to blockade of IR‐stimulated PCNA ubiquitination via apoptosis.^36^

It is worth noting that PLK1 gene depletion has also been implicated in postmitotic DNA damage as well as senescence.^66^ Moreover, BI2536 expressively induced DNA damage as well as inhibited DNA damage repair pathway in DDP‐treated cells both in vitro as well as in vivo.^67^ Intriguingly, onvansertib (OVS) augmented IR‐induced DNA damage, which was associated with γH2AX induction, suggestive of augmented DNA damage and was also consistent with the role of PLK1 in DNA damage.^68^

Remarkably, an upsurge in mitochondrial superoxide anions, general cellular ROS, activation of stress‐responsive kinases, perturbations in cell cycle progression, DNA damage, as well as apoptosis were observed after incubation of H9 NSPs in conditioned media harvested from T98G or Daoy cells.^69^ In addition, several differences in marker secretion like elevated secretion of 53BP1, total lack of p21 induction, augmented secretion of the pro‐survival protein Bcl‐2 as well as obvious resistance to IR‐induced apoptosis were detected when comparing IR‐induced DDR signaling in NSCs to fate‐restricted NPCs in the developing cerebellum in vivo to evaluate the influence of differentiation stage on the modulation of DDR signaling.^16^

Intriguingly, p21‐induced G1 growth arrest was protective from apoptosis while lack of the G1/S checkpoint in SCs was a mechanism to eliminate damaged cells as well as conserve genomic integrity.^16^ Notably, distinct DDR strategies were capable of limiting the influence of IR‐induced DSBs along the differentiation process because the pattern of genetic oncogenic changes depends on embryonic or postnatal age at IR exposure.^16^ Moreover, different DNA repair pathways, as well as other DDR mechanisms such as cell cycle checkpoint arrest as well as apoptosis functioned in a balanced fashion to adjust survival in comparison to genomic stability.^16^

## 5 SIGNALING MECHANISMS OF IRRADIATION INDUCED APOPTOSIS

In MB, the apoptotic pathways are made up of several triggers, modulators, as well as effectors.

**FIGURE 2:**
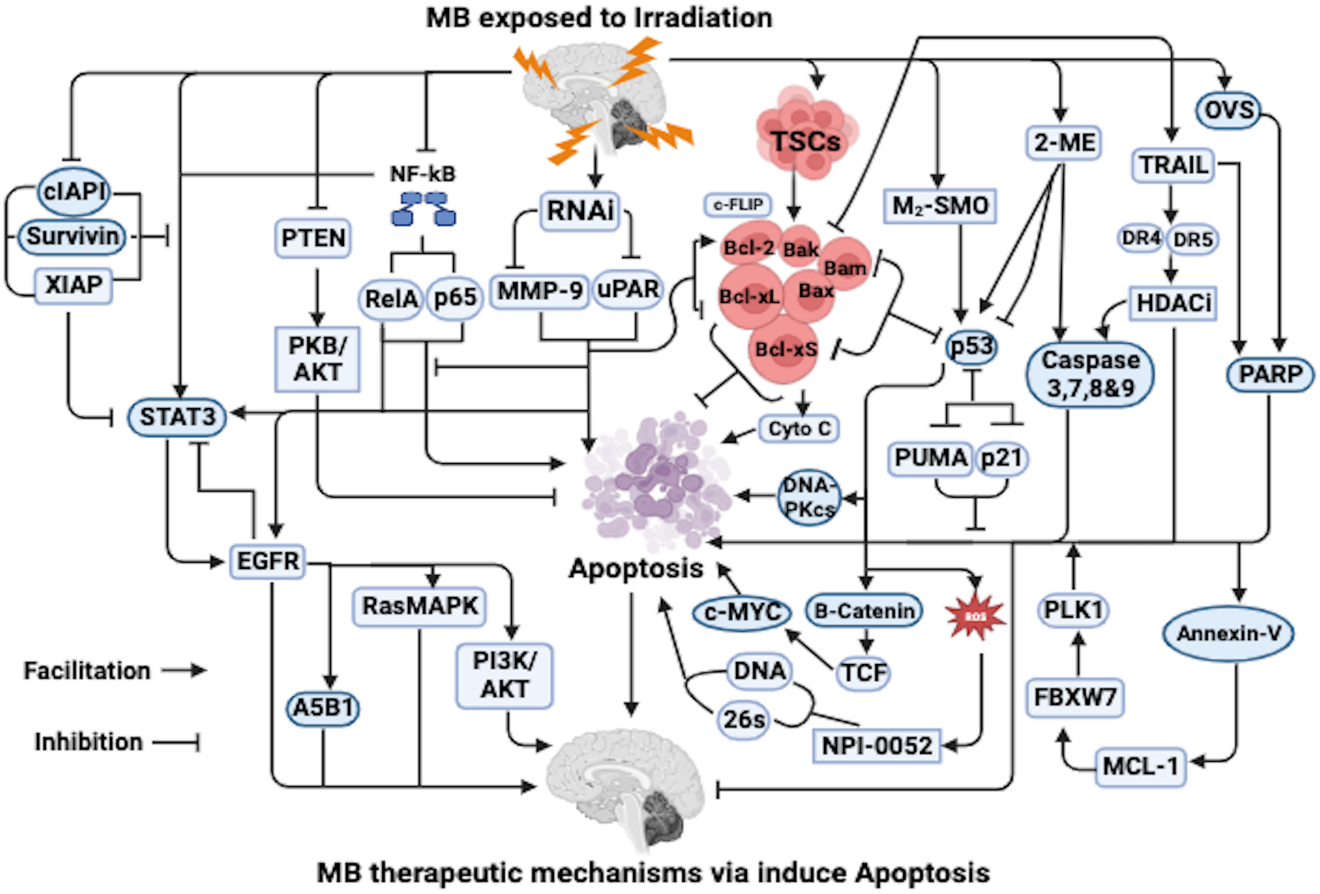
Shows the irradiation induced apoptosis therapeutic mechanisms in MB. Refer to the text for detailed explanations. Furthermore, all different variants of MB are collective term MB in the diagram. 2‐ME, 2‐methoxyestradiol; Bax, Bcl‐2‐associated X protein; Bcl2, B‐cell lymphoma 2; cIAPI, cellular inhibitor of apoptosis proteins; DNA‐PKcs, DNA‐dependent protein kinase; DR, domain‐containing receptors; EGFR, epidermal growth factor receptor; FBXW7, F‐box/WD repeat‐containing protein 7; HDACi, histone deacetylase inhibitors; MCL‐1, myeloid leukemia 1; MMP, matrix metallopeptidase; NF‐κB, nuclear factor kappa B; NPI‐0052, marizomib or salinosporamide A; OVS, onvansertib; PARP‐1, poly (ADP‐ribose) polymerase‐1; PBK, protein kinase B; PLK1, polo‐like kinase 1; PNET, primitive neuroectodermal tumors; PUMA, p53 upregulated modulator of apoptosis; Ras/MAPK, Ras/mitogen activated protein kinase; STAT, signal transducers and activators of transcription; TRAIL, TNF‐related apoptosis‐inducing ligand; TSCs, tumor stem cells; uPAR, urokinase‐type plasminogen activator receptor; XIAP, X‐linked inhibitor of apoptosis protein.

Notably, the Bcl‐2 family has been implicated in the induction of mitochondrial apoptosis which requires the involvement anti‐apoptotic gene products such as Bcl‐2, Bcl‐xL as well as pro‐apoptotic gene products such as Bax, Bak, Bcl‐xS, and Bim.^70^, ^71^ In addition, caspases which are cytosolic proteases have been implicated in the induction of apoptosis via a wide range of agents as well as extracellular signals leading to cleavage of several cellular protein substrates resulting in the impairment of tissue homeostasis and eventual destruction of the cell.^72^ Interestingly, MB and enriched MB‐tumor stem cells (TSCs) extremely secreted antiapoptotic genes such as Bcl‐2, Bcl‐xL, Bcl‐xS, Bax, Bak, Bim, c‐FLIP, and caspase 8 following IR (Figure 2).^14^, ^49^, ^68^, ^73^, ^74^

Notably, uPAR are very essential in tumor invasion as well as progression by modulating proteolysis, stimulation of other matrix proteinases, growth factors as well as triggers numerous intracellular signaling pathways.^75^, ^76^ In addition, MMPs are critical in tissue repair, tumor invasion as well as metastasis.^77^ Remarkably, IR is capable of persistent remodeling of the extracellular matrix (ECM) via the induction of proteases such as MMP‐9, uPA as well as uPAR.^49^, ^75^ Interestingly, concurrent silencing of uPAR as well as MMP‐9 using RNAi vectors was able to detect downstream signaling pathways for MB gene therapy in combination with IR via apoptosis (Figure 2).^49^

It worth noting that nuclear factor kappa B (NF‐κB) has been implicate to an antagonist to apoptosis by stimulating the secretion of anti‐apoptotic proteins as well as antioxidant molecules.^78^ Remarkably, the activity of NF‐κB depends on its nuclear translocation capacity as well as its binding to specific DNA binding sites resulting in the regulation of transcription of genes associated with cell survival as well as apoptosis.^79^ In addition, nuclear levels of the phosphorylated Rel‐A such as the p65 subunit of the NF‐κB complex as well as NF‐κB DNA binding activities were expressively blocked in uPAR as well as MMP‐9‐silenced MB cells (Figure 2).^49^ Furthermore, over secretion of NF‐κB protects cells from apoptosis, while blockade of NF‐κB induces apoptosis or sensitizes cells to IR (Figure 2).^80^

Notably, high dose x‐IR was capable of preventing early recurrence in M‐SMO tumors with normal functioning of both p53 as well as the intrinsic apoptotic pathways (Figure 2).^14^ Thus, x‐IR stimulated a survival benefit in M_2_‐SMO transgenic mice only when it triggered a p53‐dependent transcriptional response that induced apoptosis (Figure 2). It is worth noting that MB with conditional deletion of p53 were extremely resistant to x‐IR.^14^ In addition, in MB with conditional deletion of p53, x‐IR did not stimulate either the pro‐apoptotic or cell‐cycle regulatory arms of the p53‐associated transcriptional response due to lack of p53 upregulated modulator of apoptosis (PUMA) as well as p21 upregulation after x‐IR (Figure 2).^14^

Moreover, tumors with p53 deletion evaded treatment‐related apoptosis and recommenced proliferation within 24 h after treatment which was consistent with the absence of PUMA and p21 activities (Figure 2).^14^ Interestingly, MB with Bax deletion were also IR resistant, although p53 was intact with lack of apoptosis after x‐IR exposure in these tumors. Thus, it was possible to differentiate p53‐dependent effects that could otherwise have been concealed by extensive cell death.^14^, ^74^ In addition, a combination of genotoxic stress, functional p53, as well as impaired apoptosis was capable of inducing copious differentiation, as x‐IR triggered neural differentiation in tumors with intact p53 as well as Bax deletion (Figure 2).^14^, ^74^

Notably, the anti‐apoptotic Bcl‐2 signaling pathway has been implicated in resistance of NSCs to IR‐induced apoptosis in MB (Figure 2).^14^ Additionally, Bcl‐2 secretion inversely correlated with spontaneous apoptosis in patient‐derived MB samples which signify that Bax‐dependent cell death was actively regulated by other proteins (Figure 2).^14^, ^74^ It is worth noting that PTEN loss triggered stimulation of PKB (AKT) efficiently inhibited apoptosis just like Bax deletion in MB (Figure 2).^74^, ^81^ In addition, 2‐ME inhibits the growth of DAOY cells via induction of apoptosis.^1^ Furthermore, caspase 3, a key protease is triggered during the early stages of apoptosis and it is only observed in cells undergoing apoptosis (Figure 2).^1^ Interestingly, caspase 3 activity was discovered in extracts prepared from cells treated with 3 μM 2‐ME (Figure 2).^1^

Notably, 2‐ME induced caspase activity was decreased by about 64% when extracts were prepared from cells treated with Z‐VAD‐FMK, a caspase inhibitor along with 2‐ME, signifying the involvement of caspase 3 in this process.^1^ However, induced apoptosis via 2‐ME was not associated with p53 or Bax activities because there was no significant change in the levels of p53 or Bax.^1^ It is worth noting that 2‐ME induce apoptosis both through p53‐dependent as well as p53‐independent fashion cell lines (Figure 2).^82^ Interestingly, γ‐IR‐induced apoptosis which was facilitated by caspases such as ICE/CED‐3 like proteases because Z‐VAD‐FMK almost completely blocked apoptosis.^83^

Interestingly, blockade of uPAR as well as MMP‐9 reduced the secretion of Bcl‐2 and Bcl‐xL, triggered Bid cleavage, as well as augmented Bak secretion in MB cells through mitochondria derived factors mediating cell death process (Figure 2).^49^ In addition, downregulation of uPAR as well as MMP‐9 triggered high secretion of Bak with no substantial difference in Bax secretion (Figure 2).^49^ Furthermore, blockade of uPAR as well as MMP‐9 essentially augmented Bak/Bcl‐xL ratio leading to changes in the membrane potential of mitochondria which triggered the translocation of cytochrome C (Cyto C) into the cytosol and consequently resulted in the stimulation of effectors of apoptosis via mostly caspases in MB cells (Figure 2).^49^

It is worth noting that MB‐TSC cells were resistant to TRAIL because of higher secretion of Bcl‐2 and c‐FLIP in MB (Figure 2).^9^, ^73^ In addition, TRAIL was a key targets tumor cells and spares normal cells both in vitro and in vivo.^84^ Furthermore, TRAIL induces apoptosis by binding to its death domain‐containing receptors (DR) such as TRAIL‐R1/DR4 as well as TRAIL‐R2/DR5 on the cell surface resulting a cascade of caspase stimulation as well as consequent activation of the apoptotic activities.^84^, ^85^ Notably, histone deacetylase inhibitors (HDACi) act via the regulation of the epigenetic silencing as well as interrelation with TRAIL to stimulate cell death pathways in MB.^86^, ^87^ Intriguingly, HDACis' association with TRAIL occurs primarily via augmented secretion of DR5 and DR4 (Figure 2).^46^

Remarkably, TRAIL sensitivity or resistance correlated with the expression of associated DR4 and DR5 in MB cells.^9^ In addition, human mesenchymal stem cells (hMSC) S‐TRAIL induced caspase‐3 activated apoptosis in both TRAIL sensitive and resistant lines pre‐treated with an HDACi (MS‐275) in vitro and in vivo.^9^ Interestingly, MS‐275 sensitivity to MB cells were induced by TRAIL via reactivating of DR4 as well as upregulation of apoptotic caspases 3, 8, and 9, all of which were key participants in the pro‐apoptotic pathway (Figure 2).^46^ Notably, combine treatment of DAOY cells with MS‐275 and S‐TRAIL sensitized them to TRAIL‐mediated apoptosis which was observed as augmentation in apoptosis cascade such as Caspase 3 and 7 as well as cleaved PARP (Figure 2).^9^

Interestingly, silencing of uPAR as well as MMP‐9 decreased the secretion of total epidermal growth factor receptor (EGFR) in MB. Remarkably, STAT3 was fundamentally stimulated in MB via EGFR.^88^, ^89^ Notably, EGFR mediated stimulation of STAT3 translocation into the nucleus as well as triggered the transcription of genes related with cell survival following IR (Figure 2).^90^ Additionally, the EGFR/STAT3 oncogenic pathway was critical in MB tumorigenesis because it mediated cellular growth signals induced by uPAR as well as A5B1 integrins.^91^ In addition, downregulation of uPAR as well as MMP‐9 decreased the transactivation of EGFR, leading to blockade of STAT3 activation (Figure 2).^49^

Moreover, EGFR is also capable of triggering numerous intracellular signaling pathways such as Ras/mitogen activated protein kinase (Ras/MAPK) as well as phosphoinositide 3‐kinase (PI3K)/AKT.^92^ Furthermore, downregulation of STAT3 levels in MB cells lines triggered a reduction the levels of Rel‐A following IR (Figure 2).^49^ Markedly, apoptosis induced in uPAR as well as MMP‐9‐downregulated MB cells was due to inactivation of the STAT3‐related signaling pathway (Figure 2).^49^ In addition, downregulation of inhibitory apoptotic molecules like Survivin, X‐linked inhibitor of apoptosis protein (XIAP) as well as cellular inhibitor of apoptosis proteins (cIAPI) triggered blockade of STAT3/NF‐kB activity following IR (Figure 2).^93^ Interestingly, blockade of STAT3 activity triggered downregulation of Survivin following IR.^93^

Notably, OVS activated MB cell survival via apoptosis which was associated with augmentation of cleaved PARP, cleaved caspase‐3, Annexin‐V, as well as a decrease in myeloid leukemia 1 (MCL‐1).^68^ In addition, F‐box/WD repeat‐containing protein 7 (FBXW7) modulated apoptosis via MCL‐1 ubiquitination as well as destruction.^94^ Furthermore, deletion of FBXW7 mitigated PLK1 inhibitor stimulated MCL‐1 degradation.^95^ Therefore, PLK1 signaling regulates the cellular apoptosis via modulation of MCL‐1 protein stability through FBXW7 (Figure 2). It is worth noting that AKT/PKB activation was crucial for modulation stability between cell death as well as survival due to its level of phosphorylation of AKT at residue S437 following IR (Figure 2).^29^ In addition, a regulatory interaction has been implicated between PI3K‐AKT/PKB pathway and p53 following IR to the cerebellum (Figure 2).^96^

Interestingly, p53 was capable of influencing NPI‐0052's mechanism of action via augmentation of ROS concentrations in MB cells (Figure 2).^52^ Notably, p53 stabilized the cytotoxic effect of NPI‐0052 in MB cells via two parallel mechanisms.^52^ A direct mechanism in which NPI‐0052 inhibited 26S proteasome complex resulting in augmented levels of the p53 as well as an indirect mechanism via the generation of NPI‐0052‐ROS which triggered DNA damage subsequent to upregulation of p53 and finally apoptosis (Figure 2).^52^

Notably, augmentation in β‐catenin levels correlated with an augmentation in the steady‐state levels of p53 which signified an interplay between the two proteins in MB cell lines and IR augmented nuclear β‐catenin levels in these cell lines.^38^ In addition, an augmented secretion of c‐MYC, a target gene of the TCF‐β‐catenin pathway was observed, which signified that a buildup of β‐catenin in the nuclei was associated with augmented transcriptional activity.^38^ Interestingly, stabilization of β‐catenin via apoptosis was associated with growth arrest although β‐catenin nuclear buildup was related to augmented proliferation.^97^

## 6 IRRADIATION INDUCE APOPTOTIC IMMUNE MECHANISMS

Notably, all MB tumors are depicted with low quantities of infiltrating immune cells with minor disparities in the composition of immune infiltrates between the distinctive subgroups.^98^, ^99^ Remarkably, very low level of active antitumor immune responses in MB is attributed to low proportion of effector cells such like granzyme B‐expressing CD8^+^ T cells as well as natural killer (NK) cells.^100^ In addition, the quantity of T cells in MB tumors was very low compared to pediatric control tissue.^101^ Interestingly, CD4^+^ T‐cell lymphopenia was detected in MB patients who received IR and chemotherapy suggesting that this therapy affect circulating T cells in MB patients.^102^

Intriguingly, cell surface receptor molecules of the TNF/nerve growth factor (NGF) receptor superfamily such as CD95 (APO‐1/Fas) have implicated in the modulation of apoptosis.^103^ In addition, induction of CD95 via interaction of either with its specific ligand or with an agonistic antiCD95 antibody precipitously triggered apoptosis in vitro and in vivo in sensitive cells via the stimulation of death signaling cascade.^104^ Notably, CD95‐L a type II transmembrane molecule of the TNF/NGF family of ligands is expressed from cell surface via proteolytic cleavage.^105^ Interestingly, CD95‐L was upregulated in stimulated T cells leading to maintenance of homeostasis within the immune system via the removal of peripheral T‐cells following an immune response.^106^

It is worth noting that activation of the CD95 pathway was associated with γ‐IR‐induced apoptosis of MB cells.^83^ Remarkably, γ‐IR triggered apoptosis in an autocrine or paracrine manner via its associated receptor following stimulation of CD95‐L.^83^ However, modulating the interaction of CD95 ligand/receptor by inhibiting antibodies spicily decreased apoptosis. Markedly, upregulation of CD95 following a buildup of p53 in wild‐type p53 cells, but not in p53 mutant cells was observed after drug incubation and γ‐IR exposure.^107^ In addition, upregulation of CD95 was modulated by p53 at the transcriptional level or by its effect on stability of CD95 mRNA because the secretion of both CD95 mRNA as well as CD95 protein were elevated in MB cells.^83^ Thus, CD95 and its ligand/receptor interaction was functionally essential during γ‐IR‐induced apoptosis.

Remarkably, brain tumor cells secreting SC marker CD133 are relatively resistant to IR by preferential stimulation of the DNA damage response.^108^ Notably, CD133^+^ TSCs triggered MB recurrence via the STAT3 signaling axis.^109^ In addition, the number of MB CD133^+^ cells were augmented after hypoxic stimulation, and CD133^+^ cells demonstrated more radioresistant than CD133^−^ cells.^110^ Furthermore, MB‐TSC formed spheroid bodies and exhibit the extreme proportion of CD133 surface antigen under the serum free conditional culture media after IR exposure.^73^, ^111^

## 7 IRRADIATIONS INDUCE APOPTOSIS AND HORMONES

Notably, IR exposure to hypothalamic–pituitary (HP) have been implicated to induce early puberty, or, contrarywise, no or imperfect puberty due to gonadotropin deficiency.^112^, ^113^ In addition, growth hormone (GH) gonadotrophins, Adrenocorticotropic hormone (ACTH) and thyroid‐stimulating hormone (TSH) discrepancies have been observed following IR to the HP.^114^ Notably, ventromedial hypothalamus damage has been implicated in hyperphagia as well as obesity due to a direct effect on appetite control centers or due to the vagal tone disinhibition at the pancreatic beta‐cell level, resulting to insulin hypersecretion and obesity.^115^ Interestingly, insulin hypersecretion have detected in patients with obesity as result of IR.^115^

Remarkably, strong correlation between total IR dose and the development of hormonal deficiencies has been observed.^116^ Notably, low IR doses ranging from 18 to 24 Gy were capable of triggering isolated GH deficiency, whereas higher doses >60 Gy triggered multiple pituitary hormones deficiency.^116^ In addition, pituitary atrophy secondary to IR‐induced an augmented frequency as well as severity of longer‐term hormonal deficiency after IR therapy.^117^ Furthermore, GH secreting cells are the most radiosensitive, subsequent to gonadotropin‐ and adrenocorticotropin‐secreting cells and thyrotrophin is typically the last hormone affected, though alternatives may transpire in this order.^118^

Intriguingly, GH deficiency diagnosis based on the insulin‐like growth factors‐1 (IGF‐1) value reduction for the age range as well as pubertal stage.^119^ Thus, a low IGF‐1 level is highly predictive of GH deficiency, though, normal levels do not exclude this entity^120^ Notably, GH raised the serum levels of IGF‐1, which triggered cell proliferation as well as blocked apoptosis.^121^ Intriguingly, 17β‐estradiol (E2) acts as a mitogen as well as trophic factor that triggers neuronal development in the brain.^122^ In addition, both estrogen receptors such as ERα and ERβ are secreted in the developing as well as mature brain with distinctive cellular as well as developmental precises.^123^, ^124^, ^125^ Furthermore, ERβ‐like immunoreactivity was discovered in all variety of MB tumors as well as in the well‐characterized human MB cell lines D283Med and Daoy.^126^

Interestingly, physiological levels of E2 were detected in ERβ‐positive D283Med cells as well as xenografts which correlated with a dose‐dependently stimulated mitogenesis as well as tumor growth.^126^ In addition, stimulation of ERβ, but not ERα, was obligatory for the growth‐stimulatory effects of E2 signifying that estrogen responsiveness in MB was mediated by ERβ.^126^ Furthermore, residues in ERβ protein were observed as potential targets of PKCα and PKCδ which are related to ERβ and triggered its phosphorylation in Daoy cells.^127^ In addition, stimulation of ERβ triggered MB cells proliferation as well as invasion, and PKCs downregulation dysregulates these steroid receptor mediated processes. Thus, these kinases are crucial in the modulation of ERβ transcriptional activity.^125^, ^126^

It is worth noting that 2‐ME, a metabolic byproduct of estrogens and an anti‐mitotic agent is present in human urine as well as blood.^128^ Interestingly, 2‐ME was capable of blocking growth in MB cells via the induction of apoptosis.^129^ In addition, phase‐contrast microscopy as well as DNA laddering revealed that 2‐ME stimulated caspase 3 resulting in the induction of apoptosis.^1^ Moreover, 2‐ME induced apoptosis did not correlate with changes in the secretion of p53 or Bax, though, the transcriptional activity of NF‐κB promoter was decreased by 78%. Thus, induction of apoptosis was a mechanism for 2‐MEs antiproliferative activity.^1^ Currently no studies have evaluated the IR‐induce apoptosis mechanism on GH, ACTH, TSH as well as gonadotrophins in MB. Thus, further studies are needed in this direction.

## 8 IRRADIATION INDUCE APOPTOSIS THERAPEUTIC MECHANISMS

It worth noting that IR‐induced apoptotic mechanisms in MB therapy are multiplex and they either induce radiosensitivity or inhibit radioresistance leading to potential effective treatment strategies for MB. Interestingly, the occurrence of differentiation in a tumor after treatment, is not necessarily a sign of benign prognosis but rather defective apoptosis as well as augmented risk of recurrence.^14^ In addition, effective tumor treatment requires control of perivascular SCs and x‐RT was able to trigger stem cell death via intrinsic apoptotic pathway.^14^ Interestingly, IR triggered comparable apoptosis in radioresistant as well as parental DAOY cells transfected with control shRNA 48 h after x‐IR exposure.^51^ However, the parental group had expressively overwhelming apoptosis compared to the radioresistant group 72 h after IR.^51^

It was observed that a narrow time window of radiosensitivity in *Ptch* ^*neo67/+*^ cerebellum because even small age‐differences at the time of IR generated extreme modifications in MB tumorigenesis via apoptosis.^29^ In addition, MB formation in *Ptch1* mutants was related to resistance of IR‐induced cell killing.^29^ Interestingly, in MB tumors with intact p53, a single mutation in the apoptosis pathway was sufficient to trigger IR resistance via apoptosis.^34^ Furthermore, any mutation or posttranslational activity that blocked internal apoptotic pathway conferred treatment resistance in MB following IR exposure.^4^

Notably, pharmacological blockade of PKB triggered a significate reduction in cell proliferation as well as augmented apoptosis, with the reduced phosphorylation of downstream effectors of PKB such as ERK1/2 and AKT in MB following IR via apoptosis.^27^ Thus, these PKBs are potential therapeutic target in MB treatment via apoptosis. Remarkably, transcriptional as well as translational concentrations of cyclooxygenase 2 (COX‐2) were extremely upregulated in CD133^+^ cells compared to CD133^−^ cells isolated from the Daoy as well as UW228 cell lines and celecoxib augmented the response of CD133^+^ cells to high‐dose IR.^111^ Thus, the radiosensitizing effects of celecoxib on MB‐CD133^+^ cells, in vitro and in vivo, augmented radiosensitivity as well as IR‐induced apoptosis.^111^

Interestingly, therapeutic usefulness of engineered hMSCs in a xenograft mouse model of MB for a TRAIL‐sensitive cell line, as well as the sensitizing effect of HDAC inhibitor MS‐275 to stem cell‐delivered TRAIL on a TRAIL resistant cell line was established via apoptosis.^9^ Remarkably, concurrent silencing of uPAR as well as MMP‐9 using RNAi vectors acted as a potential vector for MB gene therapy in combination with IR treatment via apoptosis.^49^ In addition, PCNA function as a monoubiquitinate via E3 ligase RNF8 in IR‐induced MB cells leading to low radiosensitivity in MB cells.^49^

Furthermore, OVS in combination with IR triggered a total tumor regression of MB xenografts via apoptosis.^68^ Thus, OVS is an effective strategy for IR sensitization of Group 3 MB via apoptosis.^68^ Interestingly, interfering with the *Ptch1/SMO/SUFU/GLI* axis in the hedgehog pathway with small molecules has been implicated as a promising therapy for SHH MBs via apoptosis.^27^ Remarkably, veliparib was tested as a radiosensitizing agent in both in vitro and a mouse model of MB.^130^ It was observed a single dose of veliparib in combination with IR significantly augmented intratumoral apoptosis on immunohistochemistry staining, signifying that veliparib augmented IR induced MB cell death in vivo.^130^

Notably, knockout of RBM5‐AS1 triggered sensitization of DAOY xenograft tumors to IR, which was associated with augmented apoptosis.^51^ Thus, RBM5‐AS1 is a promising target to mitigate radioresistance in MB via apoptosis. NPI‐0052 is a second‐generation proteasome blocker that binds permanently to the 20S core subunit of the 26S proteasome.^131^ Interestingly, MB tumors secreting elevated concentrations of the 26S proteasome complex showed poor outcome in MB patients and, a combination of IR and proteasome inhibitors in inducing apoptosis in MB tumor cells.^52^ Remarkably, NPI‐0052 has a synergistic effect with γ‐IR, a constituent of the current MB therapy.^52^

Remarkably, STAT3 blockers such as LLY17 as well as LLL12B were capable of augmenting the blockade activity of IR in human MB cells via apoptosis.^53^ In addition, LLY17 as well as LLL12B suppressed cell viability as well as augmented the sensitivity of different MB subtypes to IR.^53^ Additionally, LLY17 or LLL12B with IR induces cell apoptosis and blocked STAT3 downstream proteins.^53^ Thus, combination of STAT3 blockers like LLY17 and LLL12B with IR therapy is a potential therapeutic strategy for MB. Interestingly, both Cyclin D1 and Survivin secretory levels were reduced after combination treatment with LLY17 or LLL12B with IR exposure and the induction of cell apoptosis as well as cleaved Caspase‐3 were augmented.^53^ Thus, STAT3 downstream targets were associated with cell proliferation via cyclin D1 as well as survival via Survivin.

Intriguingly, the developing cerebellum with deficient of p21 was linked to an obvious radioresistance in NSCs, whereas p21 secretion n correlates with a strong apoptotic response in fate‐restricted PCs.^16^ In addition, loss of DNA‐PKcs function blocked MB growth in the *Ptch1* ^*+/−*^ mouse model via apoptosis following IR exposure.^13^ Furthermore, combined treatment with NU7441 as well as IR reduced viability in two MB cell lines such as DAOY and D283, signifying that DNA‐PKcs might provide a potential novel therapeutic target in MB.^13^ Additionally, DNA‐PKcs blocker NU7441 radiosensitizes human MB cells in vitro via apoptosis suggesting the inclusion of MB in the list of tumors beneficiating from the combination of IR and targeting of DNA‐PKcs.^13^ Thus, DNA‐PKcs via specific inhibitor NU7441 augments radiosensitivity in human MB cell lines.

## 9 CONCLUSION

In MB, the apoptotic pathways are made up of several triggers, modulators, as well as effectors. In addition, MB tumors are depicted with low quantities of infiltrating immune cells with minor disparities in the composition of immune infiltrates between the distinctive subgroups Additionally, MB are strikingly sensitive to DNA‐damaging therapies and the role of apoptosis a key treatment modality. Furthermore, GH gonadotrophins, ACTH and TSH discrepancies have been observed following IR to the HP, however, IR‐induce apoptosis mechanism on GH, ACTH, TSH as well as gonadotrophins in MB is less explored. Interestingly, IR‐induced apoptotic mechanisms in MB therapy are complex and they either induce radiosensitivity or inhibit radioresistance leading to potential effective treatment strategies for MB.

## AUTHOR CONTRIBUTIONS

**Seidu A. Richard:** Conceptualization; methodology; writing – original draft; writing – review and editing; resources.

## CONFLICT OF INTEREST STATEMENT

The authors have stated explicitly that there are no conflicts of interest in connection with this article.

## ETHICAL STATEMENT

Not applicable.

## Data Availability

Data sharing is not applicable to this article as no new data were created or analyzed in this study.
